# Effects of Sertraline and Fluoxetine on P-Glycoprotein at Barrier Sites: *In Vivo* and *In Vitro* Approaches

**DOI:** 10.1371/journal.pone.0056525

**Published:** 2013-02-28

**Authors:** Amita Kapoor, Majid Iqbal, Sophie Petropoulos, Hay Lam Ho, William Gibb, Stephen G. Matthews

**Affiliations:** 1 Wisconsin National Primate Research Center, University of Wisconsin-Madison, Madison, Wisconsin, United States of America; 2 Departments of Physiology, Obstetrics and Gynecology and Medicine, Faculty of Medicine, University of Toronto, Toronto, Ontario, Canada; 3 University of Ottawa, Department of Obstetrics and Gynecology, Cellular and Molecular Medicine, Ottawa, Ontario, Canada; Western University of Health Sciences, United States of America

## Abstract

**Background and Purpose:**

Retention of substances from systemic circulation in the brain and testes are limited due to high levels of P-glycoprotein (P-gp) in the luminal membranes of brain and testes capillary endothelial cells. From a clinical perspective, P-gp rapidly extrudes lipophilic therapeutic agents, which then fail to reach efficacious levels. Recent studies have demonstrated that acute administration of selective serotonin reuptake inhibitors (SSRI) can affect P-gp function, *in vitro* and *in vivo*. However, little is known concerning the time-course of these effects or the effects of different SSRI *in vivo*.

**Experimental Approach:**

The P-gp substrate, tritiated digoxin ([^3^H] digoxin), was co-administered with fluoxetine or sertraline to determine if either compound increased drug accumulation within the brains and testes of mice due to inhibition of P-gp activity. We undertook parallel studies in endothelial cells derived from brain microvessels to determine the dose-response and time-course of effects.

**Key Results:**

*In vitro*, sertraline resulted in rapid and potent inhibition of P-gp function in brain endothelial cells, as determined by cellular calcein accumulation. *In vivo*, a biphasic effect was demonstrated. Brain accumulation of [^3^H] digoxin was increased 5 minutes after treatment with sertraline, but by 60 minutes after sertraline treatment, brain accumulation of digoxin was reduced compared to control. By 240 minutes after sertraline treatment brain digoxin accumulation was elevated compared to control. A similar pattern of results was obtained in the testes. There was no significant effect of fluoxetine on P-gp function, *in vitro* or *in vivo*.

**Conclusions and Implications:**

Acute sertraline administration can modulate P-gp activity in the blood-brain barrier and blood-testes barrier. This clearly has implications for the ability of therapeutic agents that are P-gp substrates, to enter the brain when co-administered with SSRI.

## Introduction

Phospho-glycoprotein (P-gp) was first discovered in mammalian tumour cells where it confers drug resistance [1]. It is a plasma membrane ATP binding cassette (ABC)-transporter of the multidrug resistance (MDR) family. P-gp can actively transport a wide range of structurally diverse xenobiotics against a concentration gradient [2]. Specific substrates include anticancer anthracyclines (doxorubicin) and alkaloids (vincristine, vinblastine), immunosuppressives (cyclosporine A), cardiac glycosides (digoxin) and steroid hormones (aldosterone, cortisol and dexamethasone) [3], [4]. P-gp is also expressed in number of normal tissues where it plays a role in absorption, distribution and excretion. It is present at the biliary canalicular surface of hepatocytes, luminal surface of cells of the jejunum and colon, the kidney and the apical membrane of the placental syncytiotrophoblast [5], [6], [7], [8]. High levels of P-gp are also found in the luminal membranes of the endothelial cells that line the microvessels of the blood-brain barrier (BBB) and blood-testis barrier (BTB) where it functions to prevent transfer of compounds from the blood into the brain and testis [9], [10], [11], [12].

The presence of drug efflux transporters in the blood capillary cells of the BBB and BTB present a major clinical obstacle: many lipophilic therapeutic agents are rapidly extruded out, and thus fail to reach efficacious levels within the brain and/or testis. This has been demonstrated in patients treated for brain or testicular tumours, epilepsy, brain HIV, Alzheimer's disease and Parkinson's disease [13], [14], [15]. Thus, the search for P-gp reversal agents has been an area of intense research [16]. The first generation candidates were found to require effective doses that exceeded their safety limits, leading to adverse side effects and toxicity. The second and third generation candidates while less toxic, have produced highly variable results in clinical trials [17], [18], [19].

Recent studies have demonstrated that the selective serotonin reuptake inhibitors (SSRI) are substrates of P-gp [20]. In L-MDR1 cells and primary porcine brain endothelial cells it was shown that sertraline, its metabolite desmethylsertraline, and paroxetine inhibited P-gp function. These effects were comparable to the potency of the classic P-gp inhibitor, quinidine [21]. Further, the cytotoxicity of vinblastine, mitomycin C, paclitaxel and doxorubicin was increased in cancer cell lines when these drugs were administered simultaneously with fluoxetine. This was also found *in vivo*, with reduced tumour volumes in mice co-administered doxorubicin and fluoxetine compared to doxorubicin alone [16], [22].

A recent study in pregnant mice has shown that sertraline increased P-gp mediated substrate efflux in the placenta, but reduced P-gp mediated substrate efflux in the maternal and fetal BBB, 4 hours after administration [23]. Therefore, the aim of the current study was to determine the time course of the inhibition of P-gp at the BBB and BTB by sertraline and fluoxetine. As such, we established the most effective dose for SSRI mediated modulation of P-gp using guinea pig primary brain endothelial cells. We then determined whether these effects could be repeated in an *in vivo* mouse model.

## Materials and Methods

### Primary Endothelial Cell Culture

Primary brain microvascular endothelial cells were isolated from 6 two-week old male guinea pigs, as been described in details previously [24]. Animals were anesthetized with isoflurane (2–3%, IsoFlo®, Isoflurane, USP, Abbott Laboratories, Limited, Saint-Laurent, Quebec) prior to euthanasia by decapitation. All animal studies were performed using protocols approved by the Animal Care Committee at the University of Toronto (Protocol: 20007062) and in accordance with the Canadian Council for Animal Care.

Brains were rapidly removed and homogenized on ice in sterile Medium 199 (Invitrogen, Carlsbad, CA) supplemented with antibiotics-antimycotics (Invitrogen). Homogenates were centrifuged at 1000 g for 5 minutes at 4°C. Pellets were resuspended in 17.5% Dextran/Hank's Balanced Salt Solution, (Sigma-Aldrich, St. Louis, MO) and centrifuged at 4200 g for 15 minutes at 4°C. The vessel fraction was digested in Collagenase I (1 mg/mL in Dulbecco's Modified Eagle Medium (DMEM); Sigma-Aldrich) for 15 minutes at 37°C. Following enzymatic digestion, the vessel/collagenase mixture was centrifuged at 500 g for 10 minutes at 4°C. The collagenase supernatant was removed and cells were resuspended in culture medium. Cells were grown to confluence in (0.5%) gelatin-coated flasks (Becton Dickinson Biosciences, Franklin Lakes, NJ), trypsinized, and re-plated in gelatin-coated 96-well plates (Becton Dickinson Biosciences). At confluence, cells were utilized for calcein accumulation assay. We have previously extensively characterized the cells derived through these isolation and culture conditions and have shown that cells stain positive for von Willebrand factor, glucose transporter 1 and zonula occludens 1, confirming endothelial phenotype [24].

#### Calcein Accumulation Assay

Calcein-acetoxymethyl (AM) is a specific substrate of P-gp and is actively extruded from brain microvascular endothelial cells. Once in the cell, calcein-AM is rapidly and irreversibly cleaved by non-specific esterases to form fluorescent calcein. Calcein, which is not a substrate of P-gp, remains in the cell. Therefore, accumulation of fluorescent calcein is used as a reliable measure of P-gp function [24].

Media was aspirated from cells grown in 96-well plates, and washed with Tyrode's Salt solution supplemented with 1 g/L sodium bicarbonate (Sigma-Aldrich, St. Louis, MO). A 200 µL Tyrode mixture containing 1 µM calcein-AM (Sigma-Aldrich) and sertraline (Sigma-Aldrich; 10^−3^–10^−8^ M), fluoxetine (Sigma-Aldrich; 10^−3^–10^−6^ M) or verapamil (Sigma-Aldrich; 10^−3^, 10^−4^ M) was added to wells. Control wells contained calcein-AM (1 µM) with no SSRI, and background was established with Tyrode solution alone. All treatments were undertaken in octuplet in 6 independent experiments with brain endothelial cells from 6 independent guinea pigs. Sertraline and verapamil treated cells were incubated for 15, 60, 120 and 240 minutes. Fluoxetine treated cells were incubated for 15 and 60 minutes. At the end of incubation, plates were put on ice to stop transfer, and washed twice with cold Tyrode solution, lysed, and accumulation of fluorescent calcein was measured using a fluorescent plate reader (Excitation/Emission: 485/510 nm; Biotek, Winooski, VT). Relative fluorescence is presented as percent control well fluorescence with background subtracted [24]. The cytotoxic effects of sertraline, fluoxetine and verapamil were assessed using trypan blue as previously described [24].

### Animal Studies

#### Animals

Male FVB mice (12–20 weeks of age) were purchased from Taconic (Germantown, NY). There were 6–9 control, 4–7 sertraline-treated and 4–7 fluoxetine treated mice used in each experimental group. Mice were housed (3–4/cage) with food and water available ad libitum.

#### Experimental Protocol

The protocol was adapted from studies previously performed in our and other laboratories [6], [25]. Mice were intravenously injected (tail vein) with either fluoxetine or sertraline (10 mg/kg) and [^3^H] digoxin (mixture: 0.05 mg/kg, unlabeled digoxin (Sigma-Aldrich) with [^3^H] digoxin 1 µCi/30 g body weight (PerkinElmer, Boston, MA)). To determine the time course of the fluoxetine effects (10 mg/kg), experiments were conducted as above, however mice were killed 5 minutes, 15 minutes, 1, 4, 12 and 24 hours after injection of fluoxetine and [^3^H] digoxin. For the sertraline time course experiment (10 mg/kg) an additional 1 minute time point was included. Control mice were injected with [^3^H] digoxin and saline to control for volume. At each time point, the number of mice in each treatment group was as follows (control, n = 6–9; sertraline, n = 4–7; fluoxetine, n = 4–7). At 1 and 5 minutes, mice were killed with isoflurane. For the remaining time points, mice were killed with an intraperitoneal injection of sodium pentobarbital (120 mg/kg, MTC Pharmaceuticals, ON). Blood was collected via cardiac puncture into tubes containing heparin, and plasma was separated by centrifugation. In addition to plasma, the brain, left and right testes, heart, a portion of the right lobe of the liver and left kidney were collected to measure accumulation of [^3^H] digoxin. Digoxin is not metabolized by the cytochrome P450 enzymes and is considered to be the benchmark substrate for assessing P-gp activity [6], [19], [25], [26].

#### Tissue Processing

Tissues were processed as described previously [25], [27]. Briefly, the brain, testes, heart, liver and kidney were homogenized in PBS (2 µL/g of tissue) and an aliquot of tissue homogenate (200 µL brain, liver and kidney; 100 µL testis and heart) and plasma (100 µL) were solubilized in SOLVABLE (PerkinElmer). Hydrogen peroxide (30%; 100 uL) was then added to decolorize samples and optimize counting efficiency prior to addition of scintillation fluid (10 mL; Ultima-Gold, PerkinElmer). Radioactivity (disintegrations per minute; DPM) was determined on a Tri-Carb Beta-Counter (PerkinElmer, address). Levels of [^3^H] digoxin are presented as a ratio of the tissue to plasma concentration to represent penetration into the tissue, or as raw DPM counts.

### Statistical Analysis

Data is presented as mean ± standard error of the mean (SEM). Data from the left and right testis were not significantly different and were averaged. Calcein uptake was analyzed using 1-way analysis of variance (ANOVA) with Dunnett's post hoc. Time data was analyzed using unpaired t-test between control and SSRI treated mice. If the variance was found to differ significantly, data was log-transformed and reanalyzed. If variance remained significantly different, the Mann-Whitney U test was used. Significance was set at p<0.05.

## Results

### *In Vitro:* Time-dependent effects of SSRI on P-gp function

**Figure 1:**
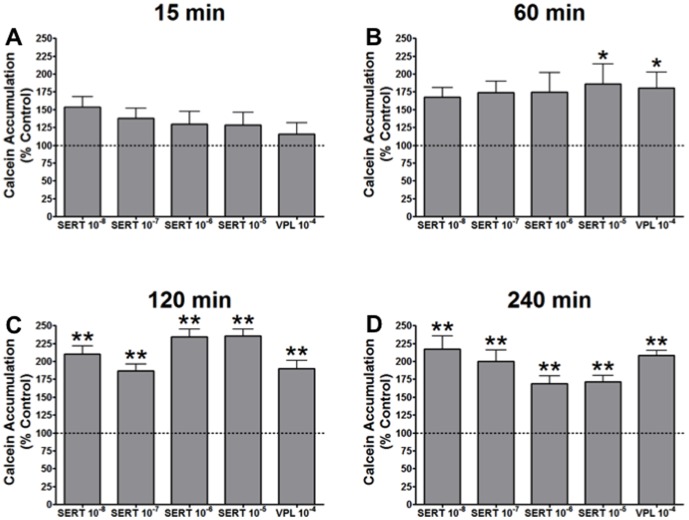
Calcein accumulation in guinea pig primary brain endothelial cells (n = 6) presented as % of control with increasing doses of sertraline (SERT) and 10^−4^ of verapamil (VPL). Calcein accumulation was assessed at (A) 15 min, (B) 60 min, (C), 120 min and (D) 240 min. * indicates p<0.05 and ** indicates p<0.01 compared to control cells.

All data are presented as the percentage of the fluorescent calcein accumulation in untreated control cells. Therefore, an increase in relative calcein accumulation represents inhibition of P-gp function. There was no significant increase in calcein accumulation at 15 minutes with sertraline treatment (Fig. 1A). At 60 minutes, sertraline treatment (10^−5^ M) resulted in an 86% increase in calcein accumulation compared to control cells (p<0.05; Fig. 1B). By 120 minutes, all doses of sertraline resulted in significant inhibition of P-gp function – peaking at a 135% increase in calcein accumulation in cells treated with 10^−5^ M sertraline (p<0.01; Fig. 1C). Likewise at 240 minutes, all doses of sertraline displayed significant inhibition of P-gp function (P<0.05; Fig. 1D).

**Figure 2:**
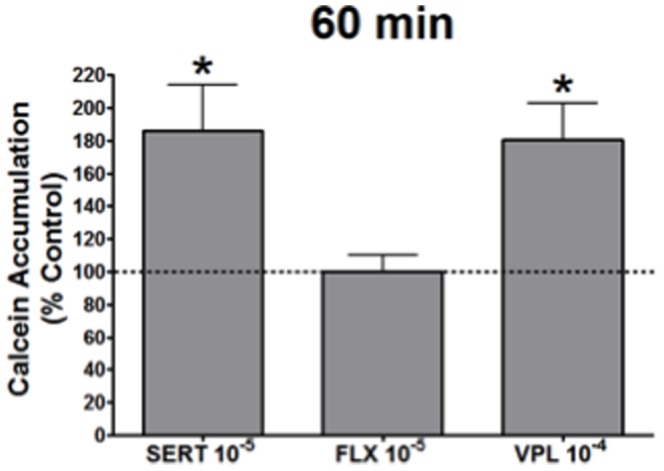
Calcein accumulation in guinea pig primary brain endothelial cells (n = 6) presented as % of control with 10^−5^ dose of sertraline (SERT), fluoxetine (FLX) and 10^−4^ of verapamil (VPL) assessed at 60 min. * indicates p<0.05 compared to control cells.

Levels of inhibition caused by sertraline treatment were comparable to the inhibition induced by treatment with the commonly used P-gp inhibitor, verapamil (Fig. 2). P-gp inhibition by verapamil (10^−4^ M) followed a similar profile to that observed with sertraline (10^−5^ M). Treatment of cells with fluoxetine (10^−5^ M) resulted in no inhibition of P-gp at any of the time points tested (Fig. 2). Higher doses of sertraline and fluoxetine (10^−3^ & 10^−4^), and verapamil (10^−3^) resulted in significant cell death (>90%; data not shown) as determined by Trypan blue staining.

### *In Vivo:* Time-dependent effects of SSRI on P-gp function

**Figure 3:**
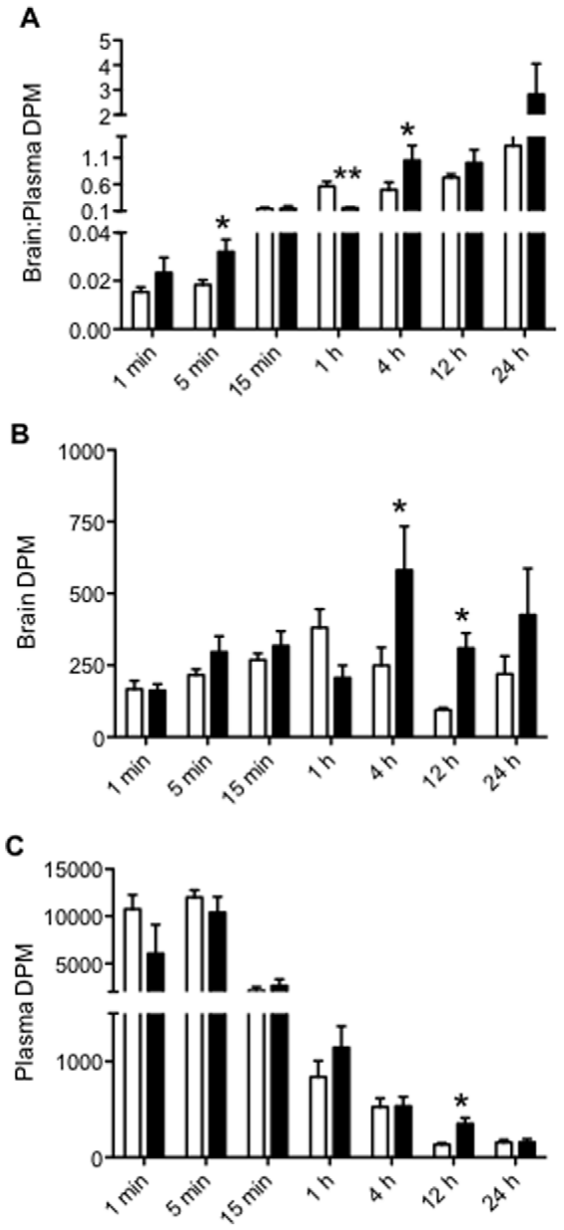
Accumulation of [3H] digoxin in FVB mice (n = 4–9/group) co-administered sertraline (10 mg/kg; closed bars) or saline (control; open bars). Mice were euthanized at various time points (1 min, 5 min, 15 min, 1 h, 4 h, 12 h, 24 h) and DPM measured in the brain, testes and plasma. All data is expressed as mean±SEM. (A) brain∶plasma DPM ratio, (B) brain DPM, (C) plasma DPM. * indicates p<0.05 vs. control. ** indicates p<0.01 vs. control.

Analysis of the brain to plasma DPM ratio revealed that [^3^H] digoxin levels were significantly higher in the brains of the sertraline treated mice at 5 minutes (Fig. 3A; p<0.05). Conversely, at 1 hour, sertraline treatment resulted in a lower brain to plasma [^3^H] digoxin ratio (p<0.01). However, by 4 hours, sertraline treated mice again demonstrated a higher brain to plasma ratio of [^3^H] digoxin (p<0.05) compared to vehicle-treated controls. Analysis of the absolute brain DPM showed that [^3^H] digoxin levels were significantly elevated in the sertraline treated mice at 4 (p<0.05) and 12 hours (p<0.01) compared to saline treated mice (Fig. 3B). Analysis of the absolute plasma DPM levels (Fig. 3C) revealed no difference between control and sertraline groups, except at 12 hours, when there was a significant elevation of plasma [^3^H] digoxin levels in sertraline-treated compared to the control mice (p<0.01).

**Figure 4:**
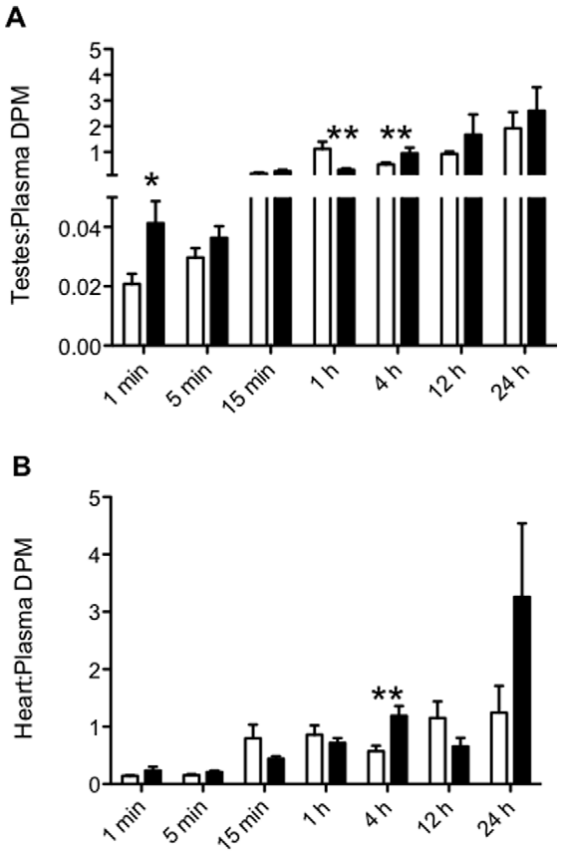
Accumulation of [3H] digoxin in FVB mice (n = 4–9) co-administered sertraline (10 mg/kg; closed bars) or saline (control; open bars). Mice were euthanized at various time points (1 m, 5 min, 15 min, 1 h, 4 h, 12 h, 24 h) and DPM measured in the testes, heart and plasma. All data is expressed as mean±SEM. (A) testes∶plasma DPM ratio, (B) heart∶plasma DPM ratio. * indicates p<0.05 vs. control. ** indicates p<0.01 vs. control.

In the testes (Fig. 4A), a very similar pattern of digoxin accumulation occurred. Sertraline treatment resulted in an increase in the testes to plasma [^3^H] digoxin ratio at 1 minute (p<0.05), a decrease at 1 hour (p<0.01) and an increase at 4 hours (p<0.01). Analysis of the heart to plasma DPM [^3^H] digoxin ratio revealed that at 4 hours the ratio was significantly higher in the mice treated with sertraline compared to control (Fig. 4B; p<0.01). There was no effect of sertraline on the liver or kidney to plasma [^3^H] digoxin ratios at any time point (data not shown). There was no significant effect of fluoxetine on accumulation [^3^H] digoxin in the brain (Table 1) or testes (Table 2) at any time point.

**Table 1:** Brain to plasma ratio (mean ± SEM) of [^3^H] digoxin in mice intravenously injected with fluoxetine (10 mg/kg) or saline (control) with digoxin (0.05 mg/kg) at different times.

Treatment	Time					
	5 minutes	15 minutes	1 hour	4 hours	12 hours	24 hours
Control	0.0187±0.0017	0.0430±0.012	0.617±0.08	0.481±0.12	1.01±0.28	1.32±0.16
Fluoxetine	0.0177±0.003	0.0339±0.012	0.426±0.076	0.262±0.071	0.987±0.60	1.79±0.33

N = 4–10 mice.

**Table 2:** Testes to plasma ratio (mean ± SEM) of [^3^H] digoxin in mice intravenously injected with fluoxetine (10 mg/kg) or saline (control) with digoxin (0.05 mg/kg) at different times.

Treatment	Time					
	5 minutes	15 minutes	1 hour	4 hours	12 hours	24 hours
Control	0.0294+0.007	0.253+0.116	1.136+0.809	0.5135+0.209	0.923+0.223	1.924+1.419
Fluoxetine	0.0334+0.005	0.360+0.002	0.101+0.019	0.360+0.053	0.739+0.575	1.280+0.382

N = 4–10 mice.

## Discussion

The aim of the current study was to determine if sertraline or fluoxetine could inhibit P-gp activity in the BBB and the BTB and, as such, facilitate the accumulation of therapeutic agents in the brain and testis, respectively. Over 24 hours, we investigated the time course of the effect. *In vitro*, sertraline treatment of primary brain endothelial cells resulted in significant inhibition of P-gp, even at the lowest dose tested (10^−8^ M). Indeed, sertraline was as potent an inhibitor of P-gp as the widely used inhibitor, verapamil. In contrast, P-gp function in brain endothelial cells was not altered by fluoxetine at any dose or time point, *in vitro*. *In vivo*, we demonstrated that there was a biphasic time-dependent effect of sertraline on the activity of P-gp at the BBB and BTB. Sertraline led to increased brain accumulation of digoxin at 5 minutes and 240 minutes (i.e. inhibited P-gp activity), but reduced accumulation of digoxin at 60 minutes (i.e. stimulated P-gp activity). An identical profile was identified at the BTB. Consistent with the *in vitro* evidence, *in vivo*, fluoxetine did not significantly affect P-gp function at the BBB or the BTB.

A recent study demonstrated that the effect of sertraline on P-gp activity is tissue-specific [23]. Simultaneous administration of [^3^H] digoxin and sertraline to pregnant mice resulted in inhibition of P-gp activity at the maternal and fetal BBB, and thus increased brain accumulation of [^3^H] digoxin. However, at the placental barrier, there was an increase in P-gp activity 240 minutes after administration. The findings in the brain are in line with the present study where we demonstrated increased accumulation of [^3^H] digoxin at 240 minutes in the brains of adult male mice. The consistency of the findings demonstrates that sertraline is an inhibitor of P-gp in endothelial cells of the brain in male, female and fetal tissues [23].

The effect of sertraline on P-gp activity at barrier sites *in vivo*, was biphasic. We demonstrated a substantial initial inhibition of P-gp by sertraline at 5 minutes in the brain and similar inhibition was identified at 1 minute in the testis. The initial effects of sertraline were highly transient at both of these barrier sites. Indeed, at 15 minutes (brain) and 5 minutes (testes) there was no difference in tissue accumulation of [^3^H] digoxin between the vehicle and sertraline treated animals. At 1 hour, sertraline-treated mice exhibited a decreased brain and testis to plasma ratio of [^3^H] digoxin compared to control, indicating that at that time, sertraline was increasing P-gp activity at both the BBB and BTB. In contrast, at 4 hours, there was once again increased accumulation within the brains and testes of sertraline treated animals, indicating inhibition of P-gp activity. One explanation for the biphasic relationship is the potential for the sertraline metabolite, desmethylsertraline, to inhibit P-gp, *in vivo*. Indeed, it has been shown *in vitro* in two cell types that the metabolite is a potent inhibitor of P-gp [21]. Future studies evaluating the effects of specific administration of desmethylsertraline *in vivo* would determine the potential of the metabolite to inhibit P-gp. Another possible explanation for the reduced digoxin accumulation in the brain at 1 h following sertraline treatment might involve changes in P-gp-independent transport of digoxin. In this connection, the organic anion transporter (Oatp1a4) has been shown to have some specificity for digoxin [28].

In the current study, there was no effect of fluoxetine on [^3^H] digoxin accumulation *in vivo*, in line with the *in vitro* results. In other studies, there have been mixed findings on the effect of fluoxetine on P-gp activity. In L-MDR1, a porcine kidney epithelial cell line transfected with the human *MDR1* gene, and porcine primary brain endothelial cells fluoxetine and its metabolites were not found to be potent inhibitors of P-gp at 60 minutes [21]. However, in tumor cells, an inhibitory effect of fluoxetine on P-gp function has been described [16], [22]. Mice co-administered doxorubicin and fluoxetine exhibited reduced tumor volumes compared to those given doxorubicin alone [16]. In another study, cytotoxicity and intracellular accumulation of doxorubicin were increased following fluoxetine treatment of cells derived from a human colorectal adenocarcinoma [22]. One possibility for the lack of an effect of fluoxetine on P-gp in the present study is the duration of treatment. Our study and those of Weiss *et al.* determined transport effects of fluoxetine over 0–240 minutes. In the studies by Peer *et al.* and Argov *et al.*, cells were incubated for 10 hours and 2 hours respectively, and mice were treated over a 3 week period [16], [22].

Of particular clinical interest is the finding that at 4 hours sertraline treatment resulted in a doubling of accumulation of digoxin in the heart. P-gp is expressed in the ventricular myocardium in human tissue. It is localized in the arterioles and capillaries where its function is to extrude substances back into circulation [29]. Consistent with our results, it has previously been found in *abcb1a/b* (−/−) mice that after 4 hours, digoxin levels were higher in the heart than wild-type controls [6]. The fact that sertraline can potently inhibit P-gp function in the heart leading to accumulation of drug substrates, may provide opportunities for improving efficacy of cardiac drugs.

Although P-gp is one of the main transporters in the brain, testis and other sites throughout the body, there are other transporters that are also functionally important. Breast cancer resistance protein (BCRP) is another ABC drug efflux protein found in various tissues such as the vascular endothelium, the epithelium of the small intestines and the proximal tubules of the kidney where it affects absorption, distribution and excretion of xenobiotics [30]. To our knowledge however, there are currently no studies that have investigated the use of fluoxetine or sertraline on BCRP activity.

In conclusion, we have shown that there is a biphasic effect of sertraline on P-gp activity at the BBB *in vivo*, and that this effect is mirrored in the BTB. The complexities of the effects of sertraline and fluoxetine in the regulation of P-gp that we have identified *in vivo*, likely underlie the inconsistencies that have emerged in the clinical use of P-gp reversal agents in clinical trials. The fact that acute treatment with sertraline can modulate P-gp function at important blood-barrier sites, *in vivo*, without causing major affects on plasma pharmacokinetics, may be utilized in the development of future therapeutics targeted at sites normally protected by P-gp and must be taken into consideration when studying drug-drug interactions.
